# High Psychiatric Morbidity and Comorbidity Among Female Prisoners in Hunan, China

**DOI:** 10.3389/fpsyt.2020.00271

**Published:** 2020-04-14

**Authors:** Shaoling Zhong, Xiaomin Zhu, Yanan Chen, Huijuan Guo, Chenyuli Luo, Xiaoxi Liang, Fanglan Wang, Hui Chen, Jiansong Zhou, Xiaoping Wang

**Affiliations:** ^1^Department of Psychiatry, The Second Xiangya Hospital, Central South University, National Clinical Research Center for Mental Disorders, National Technology Institute on Mental Disorders, Hunan Key Laboratory of Psychiatry and Mental Health, Changsha, China; ^2^Suzhou Guangji Hospital, The Affiliated Guangji Hospital of Soochow University, Suzhou, China

**Keywords:** detention, mental illness, substance use disorders, incarceration, forensic psychiatry

## Abstract

**Background:**

High prevalence of mental disorders has been found among female prisoners in Western countries, however, little is known about the epidemiology of mental disorders in such populations in China. This study aims to investigate psychiatric morbidity and comorbidity among sentenced prisoners in a female prison in China.

**Methods:**

A cluster sample of 2,703 female adult prisoners from Hunan Provincial Female Prison were interviewed with the Mini International Neuropsychiatric Interview, a semi-structured Diagnostic and Statistical Manual of Mental Disorders 4th edition (DSM-IV) mental disorder diagnostic tool. The rates of psychotic disorders, affective disorders, anxiety disorders, and substance use disorders were reported.

**Results:**

Nearly 2/3 (66.2%, N=1,790) of the sample fulfilled the criteria for at least one lifetime DSM-IV disorder 36.5% had major depression, 22.2% had post-traumatic stress disorder (PTSD), and 16.5% had drug use disorder. Drug use disorders were the major comorbid disorders. 60.8% of people with alcohol use disorder and 37.0% of those with psychotic disorders also had a drug use disorder. More than one-quarter (26.1%) of the population met criteria for a current diagnosis of any mental disorder, of which major depression was the most common (14.7%), followed by PTSD (6.4%) and psychotic disorder (1.8%).

**Conclusion:**

The high levels of psychiatric morbidity and comorbidity in a representative sample of female prisoners in China indicate unmet needs that require identification and therapeutic intervention in prisons.

## Introduction

In September 2015, the Institute for Criminal Policy Research (1) reported that there were more than 700,000 females in prison worldwide. China holds the second-largest number of female prisoners (107,131), not including those in the process of pre-trial or administrative detention. Notably, it also reported that the proportion of the prison population that is female has been growing, which has increased by 38.62% between 2005 and 2015 (1).

Higher levels of psychiatric morbidity of prisoners were identified compared to the general population in a number of studies (2–5). For example, according to a systematic review study in adult prisoners, the prevalence of psychotic illness and major depression is 3.7 and 10.5% in men and is 4 and 12% in women, respectively (2). In contrast, the prevalence of psychotic illness and major depression in the general population with similar age is 0.4 and 2.1% in the UK (3), and 0.5 and 4.6% (5) in China, respectively. The various prevalence rates suggest that prisoners have two- to ten-fold excess morbidity of psychotic illness and major depression than the general population. Some mental disorders are more prevalent in female prisoners than in male prisoners, such as posttraumatic stress disorder (PTSD), major depression, and psychotic disorders. Fazel and colleagues (6) systematically reviewed severe psychiatric morbidity in 33,588 prisoners across 24 countries and found the prevalence of major depression and psychosis was 14.1 and 3.9% in female prisoners respectively, while for male prisoners the rates were 10.2 and 3.6%. Baranyi and colleagues (7) reported a pooled point prevalence of PTSD of 21.1% for female prisoners and 6.2% for male prisoners in a most recent meta-analysis.

Undoubtedly, mental disorders are associated with a range of adverse events in prison, including self-harm (8), suicide (9), and prison violence (10). Furthermore, the presence of comorbidity was associated with these severe adverse outcomes (11). However, only some specific comorbid disorders have been reported in a body of evidence in correctional settings. For example, previous evidence showed around 20 to 40% of individuals with any mental disorder were found to have a comorbid substance use disorder (6), while limited evidence has been reported on other mental disorders (12).

The majority of evidence about mental health in female prisoners comes from Western developed countries while few have been made in developing countries (13). Fazel and colleagues’ systematic review found only nine studies from non-Western countries among 81 publications (6), without any data from China; The Baranyi review (7) identified only one study from China, which merely reported the prevalence of PTSD from a sample of 471 female prisoners (14). Furthermore, none of the studies presented comorbidity among female prisoners in China. Due to diverging economic status and public-health systems, it is uncertain to what extent the findings in Western countries can be applied to low-middle income countries.

In addition, although Mental Health Law of China which implemented in 2013, prescribes that prisons shall be concerned about the incarcerated population’s psychological well-being, and provide psychological counseling and guidance when necessary (15), the lack of mental health service in correctional settings in China is still critical. In particular, most of the prison systems are not equipped with mental health wards or professionals (16). Therefore, to assess and scale up national-specific mental health service in female prisons, epidemiological evidence is in urgent need. Thus, the purpose of this study is to investigate the prevalence and comorbidity of mental disorders in a cluster sample of female prisoners in Hunan, China.

## Methods

### Study Setting and Design

This cluster sampling cross-sectional study was carried out in Hunan Provincial Female Prison from December 1st, 2012 to December 30th, 2013. In China, almost every province has only one female prison. Hunan province is located in the central south of China, with a total population of 67 million. It is a medium-developed and representative province among the 34 provinces or administrative area in China.

### Participants

All participants were assured of confidentiality and informed that they could withdraw at any time of the study without any adverse consequences or punishment. The inclusion criteria were female prisoners who were (1) fluent in the Chinese language, (2) able to comprehend the interview questions, (3) age >= 18 years, and (4) willing to participate in the study. Those who agreed to take part in this study provided written informed consent. The study was approved by Ethics Committees of the Second Xiangya Hospital and Hunan Provincial Female Prison and Hunan Prison Administrative Bureau.

### Assessment Tools

Data on the demographic and criminal characteristics were obtained using a self-developed standard form, including ages, education levels, ethnicity, place of residence, employment status, marital status, income prior to prison, history of offence, index offence, length of sentence. The history of seeking help due to mental health problem was obtained by the following questions: In your lifetime (1) Have you ever gone to anyone or anywhere for help about your mental distress; (2) Have you been admitted to a psychiatric hospital/ward?

The diagnostic assessment of lifetime and current mental disorders was conducted using the MINI-mini International Neuropsychiatric Interview (17). MINI is a diagnostic structured interview to establish Diagnostic and Statistical Manual of Mental Disorders 4th edition (DSM-IV) diagnoses. It has been validated and applied in numerous studies in the prison population (18–20). The Chinese version of MINI has been validated in 2009 with appropriate reliability and validity (21). The diagnoses to be considered include psychotic disorders, affective disorders (major depression, dysthymia, [hypo]mania), anxiety disorders (generalized anxiety disorder, GAD; obsessive-compulsive disorder, OCD; and PTSD), and substance use disorders, including alcohol use disorder (alcohol abuse or dependence) and drug use disorder (drug abuse or dependence). The MINI generates DSM-IV diagnoses for lifetime and current-time intervals. For the prison setting, we adapted the past-12 months diagnoses of SUD into lifetime intervals. We also added the lifetime diagnoses of anxiety disorders.

### Data Collection

Prisoners who agreed and were eligible for the study completed the survey on their demographic and criminal characteristics in a quiet room. Prisoners were interviewed alone by trained psychiatrists using the MINI. Each interview took about 30 to 45 min. All interviewers were well-trained before the survey and turned to perform satisfactory inter-rater reliability. The kappa value ranged from 0.86 to 0.95 has been described and reported in our previous publication (22).

### Statistical Analysis

The socio-demographic characteristics included age, ethnicity (Han/tujia/ethnicity/others), place of residence (urban/rural), marital status (married/unmarried), and employment prior to incarceration (unemployment and employed). Education level was classified as low (illiterate and Grade 1–6), medium (Grade 7–12), and high (Grade 13 and above). Monthly Income ≤ poverty threshold (¥2,300 = $378.9 per person per year in 2013 in China was defined as low, monthly income > poverty threshold and ≤ per capita disposable income (¥23,414 = $3,807 per person per year in 2013 in Hunan) was defined as medium, monthly income > per capital disposable income was defined as high. We divided index offences into violent (homicide, assault, robbery, abduction, and arson), drug and non-violent offence (theft, fraud, bribery, etc). Length of sentence duration was classified as less than 12 months, 12 to 60 months, 60 to 120 months, 120 to 240 months and life sentence.

All data were analyzed in the IBM Statistical Package for Social Sciences (SPSS Inc., Chicago, IL, USA) version 18.0. Categorical variables were reported as numbers and percentages, and continuous variables with normal distribution were present as mean and standard deviance (sd.). Lifetime and current prevalence of mental disorders were described using percentages and 95% confidence intervals (95% CIs). Chi-square tests were used to test the difference in various demographic characteristics (i.e. age group, education level, marital status, and employment status) for each category of mental disorders. All statistical tests were considered two-sided and a value of 0.05 was considered as statistically significant.

## Results

### Demographic and Criminological Characteristics

Among 2,917 female prisoners, 207 were excluded for various reasons—161 prisoners were released during the study period, 21 refused to interview, 14 were in hospitals because of physical diseases, 8 had difficulties in hearing or talking, 2 were released on parole, and 1 was incarcerated. A total of 2,703 female prisoners were included in this study, leaving the participation rate of 92.9% (22, 23). **Table 1** lists the demographic and criminological characteristics of the sample. Participants were from 18 to 81 years old, with a mean age of 39.9 (sd:10.3) years. The majority of the sample were of Han ethnicity (90.8%), living in the urban area (68.1%), married (43.2%), having a medium level of income (60.6%), and receiving a medium level of education (55.9%). Most of them were sentenced to more than 120 months (49.0%).

**Table 1:** Demographic and criminological characteristics among the female prisoners (n=2,703).

Variables	Categories	Numbers	Percentages (%)
Education ^a^			
	Low	999	37.0
	Medium	1,510	55.9
	High	194	7.2
Ethnicity			
	Han	2,453	90.8
	Tujia ethnicity	98	3.6
	Miao ethnicity	69	2.5
	Others	83	3.1
Residence			
	Urban	1,840	68.1
	Rural	863	31.9
Employment			
	Unemployed	1,162	43.0
	Employed	1,541	57.0
Marriage			
	Single	465	17.2
	Cohabitation	108	4.0
	Married	1,170	43.2
	Divorce	702	26.0
	Widowed	258	9.5
Income^b^			
	Low	66	2.5
	Medium	1,639	60.6
	High	998	36.9
Previous offence^c^			
	No	2,520	93.2
	Yes	183	6.8
Family drug use history			
	No	2,497	92.4
	Yes	206	7.6
Family alcohol use history			
	No	982	36.3
	Yes	1,721	63.7
Family criminal history			
	No	2,332	86.1
	Yes	377	13.9
Family psychotic diseases history			
	No	2,540	94.0
	Yes	163	6.0
Index Offence^c^			
	Violent^d^	844	31.2
	Drug	1,003	37.1
	Non-violent^e^	856	31.7
Sentence group ^c^	(months)		
	0–12	33	1.2
	13–60	727	26.9
	61–120	620	22.9
	121–240	589	21.8
	lifetime	734	27.2

a Education level ≤ 6 years was defined as low, 6 years < education level ≤ 12 years was defined as medium, education level > 12 years or college was defined as high.

b Monthly Income ≤ poverty threshold (¥2,300 = $378.9 per person per year in 2013 in China) was defined as low, poverty threshold < monthly income ≤ per capita disposable income (¥23,414 = $3,807 per person per year in 2013 in Hunan) was defined as medium, monthly income > per capital disposable income was defined as high.

c Based on self-report.

d Violent offence includes homicide, assault, robbery, abduction, and arson.

e Non-violent offence includes theft, fraud, bribery, etc.

### Morbidity of Mental Disorders

**Table 2** shows the lifetime and current prevalence of mental disorders among female prisoners. In the whole sample, major depression, PTSD, and psychotic disorder were the three most common mental disorders. Among these prisoners, 66.2% (N=1,790) were diagnosed with lifetime mental disorders, with affective disorders having the highest prevalence (37.1%). Strikingly, 36.5% were diagnosed with major depression, 1.5% with dysthymia disorder, and 0.8% met the diagnostic criteria for hypomania or manic episode. Of the whole sample, anxiety disorder was second-common mental disorder. PTSD, general anxiety disorder (GAD), and obsessive-compulsive disorder (OCD) were diagnosed in 22.2, 1.6, and 0.3% of prisoners respectively. Drug dependence and drug abuse were seen in 16.5 and 5.4% respectively, and alcohol dependence and alcohol abuse were 1.7 and 3.4% respectively. Current diagnosis of any mental disorder was diagnosed in 26.1% of participants, of which major depression was the most common (14.7%), followed by PTSD (6.4%) and psychotic disorder (1.8%).

**Table 2:** Mental disorders among the female prisoners.

	Total samples (n=2703)			
	Lifetime prevalence % (95% CI)		Current prevalence % (95%CI)	
Any Axis I disorder	66.2	(64.4–68.0)	26.1	(24.5–27.8)
Psychotic disorder	3.7	(3.0–4.4)	1.8	(1.3–2.4)
Any affective disorder	37.1	(35.3–39.0)	16.4	(15.0–17.7)
Major depression	36.5	(34.7–38.4)	14.7	(13.3–16.0)
Dysthymia	1.5	(1.0–1.9)	1.5	(1.0–1.9)
(Hypo)manic	0.8	(0.4–1.1)	0.6	(0.3–0.8)
Any anxiety disorder	24.1	(22.5–25.7)	8.3	(7.2–9.3)
GAD	1.6	(1.1–2.0)	1.6	(1.1–2.0)
OCD	0.3	(0.1–0.5)	0.3	(0.1–0.5)
PTSD	22.2	(20.7–23.8)	6.4	(5.5–7.3)
Any substance use disorder	24	(22.4–25.6)	–	–
Alcohol Dependence	1.7	(1.2–2.2)	–	–
Alcohol Abuse	3.4	(2.8–4.1)	–	–
Drug Dependence	16.5	(15.1–17.9)	–	–
Drug Abuse	5.4	(4.6–6.3)	–	–

GAD, generalized anxiety disorder; OCD, obsessive-compulsive disorder; PTSD, post-traumatic stress disorder.

### Ever Sought Medical Help

When asked whether they had ever sought help for mental distress, 25.7% of the prisoners reported “yes.” They usually turned to friends, relative, clinicians, and other people for help. Only 4.7% (N=127) ever sought medical help. Among those with any mental disorder, only 5.5% (N=99) had ever sought medical help, ranging from 3.2% (drug use disorder) to 13.0% (psychotic disorder). Only 1.7% (N=30) had been hospitalized among those with any mental disorder, ranging from 0.7% (SUD) to 6% (psychotic disorder).

### Comorbidity of Mental Disorders

Comorbidities of lifetime mental disorders are listed in **Table 3**. Affective disorder and drug use disorder were the main comorbid disorders across diagnostic categories. Affective disorders are common in those with PTSD, alcohol use disorder, and drug use disorder. Nearly 45.4% (95% CI: 41.5–49.4%) of prisoners with PTSD, 40.0% (95% CI: 32.0–48.0%) of those with alcohol use disorder, and 35.2% (95% CI: 31.4–39.1%) of those with drug use disorder also met the criteria of affective disorders. Drug use disorder is also common in those with a psychotic disorder, that 37.0% (95% CI: 27.5–46.5%) of those with psychotic disorders and 60.8% (95% CI: 52.7–68.7%) of those with alcohol use disorder also had a drug use disorder.

**Table 3:** Comorbidity in mental disorders among the female prisoners.

Diagnosis	Cases with comorbid mental disoeders, % (95% CIs)									
	Psychotic disorders		Affective disorders		PTSD		AUD		DUD	
Psychotic disorders	–	–	2.0	(0–4.7)	13.0	(6.4–19.6)	9.0	(3.4–14.6)	37.0	(27.5–46.5)
Affective disorders	0.2	(0–0.5)	–	–	27.2	(24.5–30.0)	5.6	(4.2–7.0)	20.8	(18.3–23.3)
PTSD	2.2	(1.0–3.3)	45.4	(41.5–49.4)	–	–	6.0	(4.1–7.9)	16.8	(13.8–19.8)
AUD	6.4	(2.4–10.4)	40.0	(32.0–48.0)	25.7	(18.6–32.9)	–	–	60.8	(52.7–68.7)
DUD	6.2	(4.3–8.2)	35.2	(31.4–39.1)	17.0	(14.0–20.1)	14.3	(11.5–17.1)	–	–

PTSD, post-traumatic stress disorder; AUD, alcohol use disorder; DUD, drug use disorder.

### Characteristics of Prisoners With Mental Disorders

**Table 4** shows the demographic correlates for lifetime prevalence of psychotic disorders, affective disorders, PTSD, alcohol use disorder, drug use disorder, and any mental disorders. It suggests that female prisoners who were less than 45-years-old ($\chi_{1}^{2}$=70.78, p < 0.01), unmarried ($\chi_{2}^{2}$=49.60, p < 0.01), and unemployed ($\chi_{2}^{2}$=49.60, p < 0.01) were more likely to have suffered from any mental disorder than the others.

**Table 4:** Demographic correlates for lifetime prevalence of psychotic disorders, affective disorders, post-traumatic stress disorder, alcohol use disorder, drug use disorder, and any mental disorders among the female prisoners.

Variables		Psychotic disorders	Affective disorders	PTSD	AUD	DUD	Any mental disorder
		% (95% CIs)	% (95% CIs)	% (95% CIs)	% (95% CIs)	% (95% CIs)	% (95% CIs)
Age (yrs)	< 45	4.6 (0.5–8.7)	39.8 (36.8–42.8)	21.5 (18.2–24.8)	6.1 (2.1–10.1)	28.6 (25.0–32.2)	70.2 (68.1–72.3)
	45–59	1.3 (0–3.5)	31.2 (28.3–34.1)	24.8 (21.3–28.3)	3.3 (0.3–6.3)	8.7 (6.4–11.0)	57.6 (55.3–59.9)
	> 59	5.0 (0.7–9.3)	33.7 (30.8–36.6)	15.8 (12.9–18.7)	3.0 (0.2–5.8)	0.0 (0.0–0.0)	59.4 (47.9–70.9)
		$\chi_{2}^{2}$=17.23^***^	$\chi_{2}^{2}$=17.50^***^	$\chi_{2}^{2}$=5.86	$\chi_{2}^{2}$=9.90^**^	$\chi_{2}^{2}$=154.50^***^	$\chi_{2}^{2}$=40.76^***^
Education^a^	Low	2.7 (0.0–5.9)	40.6 (37.6–43.6)	24.4 (21.0–27.8)	3.3 (0.3–6.3)	13.8 (11.0–16.6)	65.8 (63.6–68.0)
	Medium	4.3 (0.3–8.3)	34.4 (31.5–37.3)	20.4 (17.2–23.6)	6.8 (2.6–11.0)	29.0 (25.3–32.7)	66.8 (64.6–69.0)
	High	4.1 (0.2–8.0)	40.2 (37.2–43.2)	25.3 (21.8–28.8)	2.6 (0.0–5.2)	8.8 (6.5–11.1)	64.4 (62.2–66.6)
		$\chi_{2}^{2}$=4.34	$\chi_{2}^{2}$=10.99^**^	$\chi_{2}^{2}$=6.74^*^	$\chi_{2}^{2}$=17.47^***^	$\chi_{2}^{2}$=102.22^***^	$\chi_{2}^{2}$=0.56
Marital status	Married	2.6 (0–5.7)	33.7 (30.8–36.6)	20.8 (17.6–24.0)	4.0 (0.8–7.2)	14.3 (11.5–17.1)	58.9 (56.6–61.2)
	Unmarried	4.5 (0.4–8.6)	39.7 (36.7–42.7)	23.4 (20.0–26.8)	6.1 (2.1–10.1)	27.8 (24.2–31.4)	71.8 (69.7–73.9)
		$\chi_{1}^{2}$=6.58^*^	$\chi_{1}^{2}$=10.41^***^	$\chi_{1}^{2}$=2.56	$\chi_{1}^{2}$=5.68^*^	$\chi_{1}^{2}$=70.78^***^	$\chi_{2}^{2}$=49.60^***^
Employment	No	4.7 (0.6–8.8)	36.7 (33.7–39.7)	20.4 (17.2–23.6)	6.5 (2.4–10.6)	35.0 (31.2–38.8)	71.9 (69.8–74.0)
	Yes	2.9 (0–6.2)	37.4 (34.4–40.4)	23.6 (20.2–27.0)	4.2 (0.9–7.5)	12.1 (9.5–14.7)	62.0 (59.8–64.2)
		$\chi_{1}^{2}$=6.11^*^	$\chi_{1}^{2}$=0.11	$\chi_{1}^{2}$=3.99^*^	$\chi_{1}^{2}$=6.75^**^	$\chi_{1}^{2}$=203.85^***^	$\chi_{2}^{2}$=28.95^***^

PTSD, post-traumatic stress disorder; AUD, alcohol use disorder; DUD, drug use disorder. ***p < 0.001, **p < 0.01, *p < 0.05.

^a^Education level < 6 years was defined as low, 6 years < education level ≤ 12 years was defined as medium, education level > 12 years or college was defined as high.

Diagnosis of psychotic disorders, affective disorders, alcohol use disorder, and drug use disorder were statistically significant associated with age group ($\chi_{2}^{2}$=17.23, p < 0.01; $\chi_{2}^{2}$=17.23, p < 0.01; $\chi_{1}^{2}$=9.90, p=0.007; and $\chi_{2}^{2}$=154.50, p < 0.01; respectively) and not married ($\chi_{1}^{2}$=6.58, p=0.012; $\chi_{1}^{2}$=10.41, p < 0.01; $\chi_{1}^{2}$=5.68, p=0.017; $\chi_{1}^{2}$=70.78, p < 0.01; respectively). Female prisoners with medium education level (6–12 years) were more likely to have suffered from alcohol use disorder ($\chi_{2}^{2}$=17.47, p < 0.01) and drug use disorder ($\chi_{2}^{2}$=102.22, p < 0.01), but less likely to have affective disorders ($\chi_{2}^{2}$=102.22, p=0.004) than the others. Also, a higher proportion of prisoners who were not employed suffered from psychotic disorder ($\chi_{1}^{2}$=6.11, p=0.013), alcohol use disorder ($\chi_{1}^{2}$=3.99, p=0.09), and drug use disorder ($\chi_{1}^{2}$=203.85, p < 0.01), while those who were employed were more likely to have PTSD ($\chi_{1}^{2}$=3.99, p=0.046).

## Discussion

To our knowledge, this is the first study to investigate the prevalence and comorbidity of mental disorders using a structured assessment tool in female prisoners in China. There are three main findings from this study. First, two-thirds of female prisoners had a lifetime diagnosis of any mental disorder. Major depression was the most common lifetime mental disorder, followed by PTSD and drug dependence. Over one-quarter of prisoners met diagnostic criteria for any current mental disorder. Major depression had the highest prevalence in current diagnosis and was found in 14.7%. Second, drug use disorders were major comorbid disorders, especially in alcohol use disorder and psychotic disorders. Third, a number of demographic characteristics were correlated with a lifetime prevalence of psychotic disorders, affective disorders, PTSD, alcohol use disorder, drug use disorder, and any mental disorder. Prisoners with different demographic and socioeconomic characteristics had a diverging prevalence of mental disorders. The morbidity and comorbidity of mental disorder in female prisoners reinforced the burden of mental disorders and the need for mental health care in correctional settings.

Similar to previous findings in Western countries (2, 6, 24, 25) and non-Western countries (12), the prevalence of mental disorders in this study is much higher compared to the general population (5, 26). Specifically, a large-scale psychiatric survey including 63,004 individuals conducted by Phillips and colleagues (26) reported the adjusted 1-month prevalence rate of psychotic disorder, mood disorder, and anxiety disorder was 0.9, 7.3, and 7.3% respectively among the female population in China. More recently, the nationwide mental epidemiological survey (5), conducted between July 2013 and March 2015, reported the 1-month prevalence in female individuals of psychotic diseases, mood disorder, anxiety disorder, and substance use disorder as 0.5, 4.6, and 5.2% respectively. This implies the prevalence of mental disorders in female prisoners is about two to three times that in the general female population. The variance may be partially explained by imprisonment itself and the specific environment of confinement, such as being separated from their families (27), which were reported to substantially increase the risk of mental disorders in the prison setting. It may also link to a lack of prison mental health service and insufficient psychiatric care (28).

When compared to other female prison populations, the prevalence of any lifetime and current mental disorders in this study was higher than in a previous study conducted in southeast China in 2011, which reported the lifetime prevalence as 22.5% and current prevalence as 12.8% (29). The difference observed may be explained by study design and classes of mental disorders. In detail, the previous study used stratified sampling, while we applied cluster sampling. Further, some common mental disorders (e.g. PTSD, GAD) were not screened in the previous study. These common mental disorders were prevalent in female prisoners (14), which may partly explain a higher prevalence in our study than the previous.

The prevalence of major depression in female prisoners identified in this study is similar to previous findings in a meta-analyses study (ranged from 10.2 to 18.1%) (6). Our findings are similar with the previous study from China (29), which reported the point prevalence of psychotic disorder and lifetime prevalence of schizophrenia as 1.83% (95% CI: 127–2.39) and 2.24% (95% CI: 1.62–2.86) respectively. The prevalence of a psychotic disorder in our study was also in line with a recent meta-analysis, which included 14,527 prisoners from 13 low-and middle-income countries and the pooled 1-year prevalence of a psychotic disorder in female prisoners varied from 1.9 to 11.0% (30).

Our findings appear to have a higher lifetime PTSD diagnosis rate but a lower current diagnosis rate than a previous study conducted in China by Huang et al. (14). In the survey, they included 471 female prisoners in Hunan female prison in 2004 and found the prevalence of lifetime and current PTSD were 15.9 and 10.6% respectively. But similar to Huang’s study, both lifetime and current prevalence of PTSD in our study are lower than studies in Western countries (7, 31, 32). The prevalence of drug use disorder and alcohol use disorder in this sample appears to be higher than the survey in Guangxi (29), but lower than in Western studies (ranged from 10 to 24%, and 30 to 60% in alcohol use disorder and drug use disorder, respectively) (24, 32, 33). These may be explained by various screening or diagnostic tools [the Composite International Diagnostic Interview (32, 33), vs. M.I.N.I], sampling [random sampling (32, 33) vs. cluster sampling] and different sampled population.

### Clinical Implications

Overall, the psychiatric morbidity and comorbidity indicated a substantial burden among female prisoners in China. There are two main implications of this study. First, the results highlight the need for screening for mental disorders at prison admission. Second, effective interventions are required to meet the need of prisoners with psychiatric morbidity, especially for those with comorbidity. Despite the fact that investigating effective prison health services to improve the mental health state of female prisoners seems reasonable (34), inadequate efforts from the government have been put to meet the needs (35, 36). Beyond clinical works, comprehensive mental health actions require targeting the root causes of female prisoners’ distress. Specifically, limited prison-based mental health resources have been put in China (16, 37). Therefore, we advocate that resources and investigations should be put into improving mental health care in the prison context.

### Limitations

The study also has several limitations. Firstly, using cluster sampling, the study was conducted in the solely female prison in Hunan province, the sample is not necessarily representative of the whole prison population in China. It must be cautious when generalizing the findings to male prisons or other areas. Secondly, two prisoners presenting with obvious symptoms of psychosis or accurate episode of another severe mental disorder were transferred out of the prison during the study period. These prisoners were not able to be included in this study, thus the prevalence of mental disorders might be underestimated. However, our findings suggested the prevalence of mental disorders among those who are inside the wall remain a major concern. Finally, the information of criminal characteristics was self-reported, therefore recall bias may exist.

### Conclusion

In conclusion, two-thirds of prisoners had a lifetime diagnosis of mental disorders and one-quarter of prisoners met a current diagnosis of mental disorder. Comorbidity is common and suggests a high burden of mental disorder among female prisoners. This highlight the importance of screening mental disorders in prison-based services, more developing effective strategies to identify and treat female prisoners with mental disorders.

## Data Availability Statement

The datasets generated for this study are available on request to the corresponding authors.

## Ethics Statement

The studies involving human participants were reviewed and approved by Ethics Committees of the Second Xiangya Hospital. The patients/participants provided their written informed consent to participate in this study.

## Author Contributions

SZ contributed to the conception, analysis, and drafting of the manuscript. XW and JZ were responsible for the study design, interpretation of the data, and revising the manuscript. XZ was involved in the study design and acquisition of the data. YC, HG, CL, XL, FW, and HC were engaged in the acquisition and analysis for the work. All authors provided approval for publication of the content.

## Funding

This study was by the National Key Research and Development Program of China (2016YFC0800701), National Natural Science Foundation of China (81571341), and Natural Science Foundation of Hunan, China (2019JJ40424) and the Natural Science Foundation of Jiangsu Province (BK20180213), and the Fundamental Research Funds for the Central Universities of Central South University.

## Conflict of Interests

The authors declare that the research was conducted in the absence of any commercial or financial relationships that could be construed as a potential conflict of interest.
